# Supplementary material for: DMRforPairs: identifying Differentially Methylated Regions between unique samples using array based methylation profiles
Source: BMC Bioinformatics. 2014 May 15;15:141. doi: 10.1186/1471-2105-15-141 (PMC4046028; doi:10.1186/1471-2105-15-141)
Supplement: Additional file 2 — DMRforPairs output for the comparison of A431-MCF7 and NA17018-NA17105. Please start from the HTML files in each folder. Available via the BMC Bioinformatics website. [file 1471-2105-15-141-S2.zip › 1394847754114233_MOESM2_ESM/A431_MCF7/figures/14986.pdf]

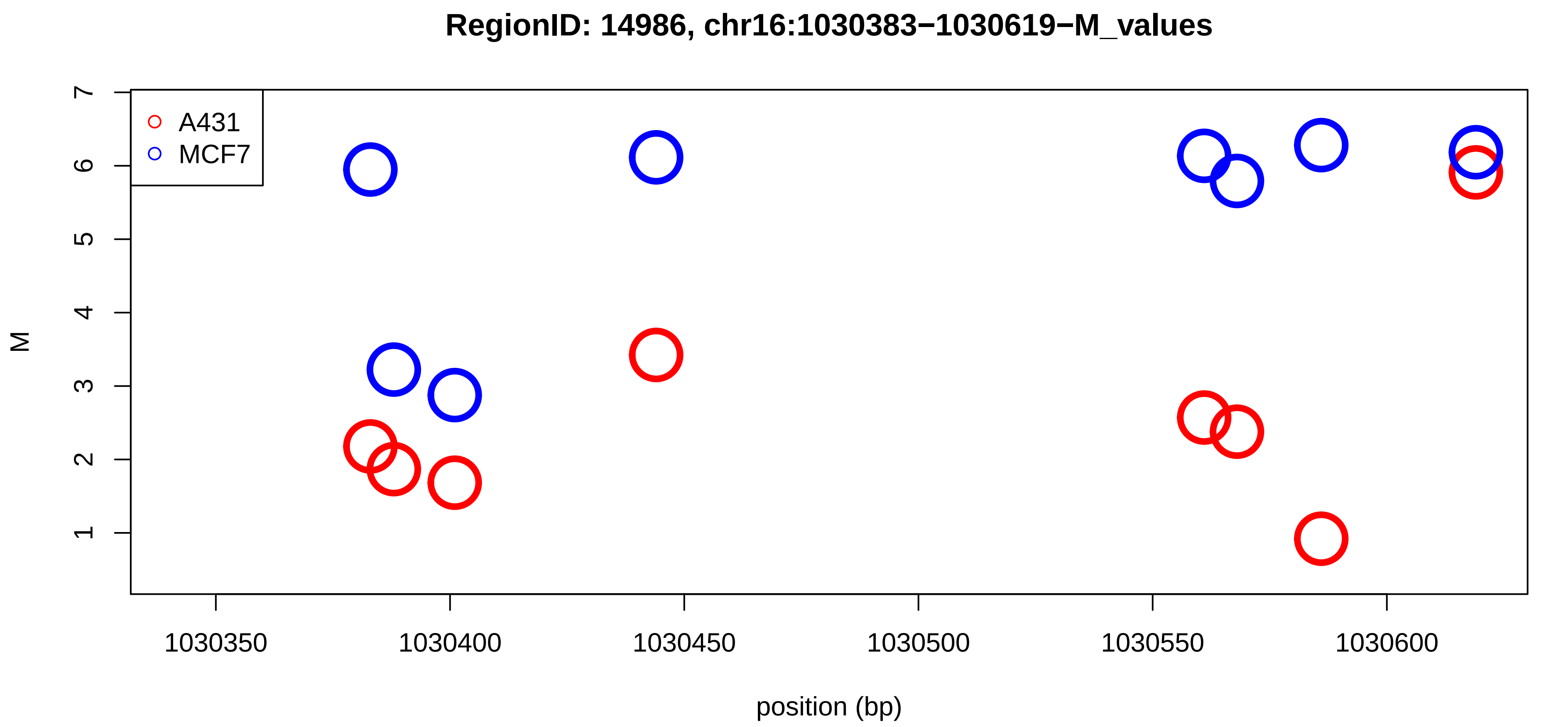

RegionID: 14986, chr16:1030383−1030619−M_values
A431
MCF7
M
1
2
3
4
5
6
7
1030350
1030400
1030450
1030500
1030550
1030600
position (bp)

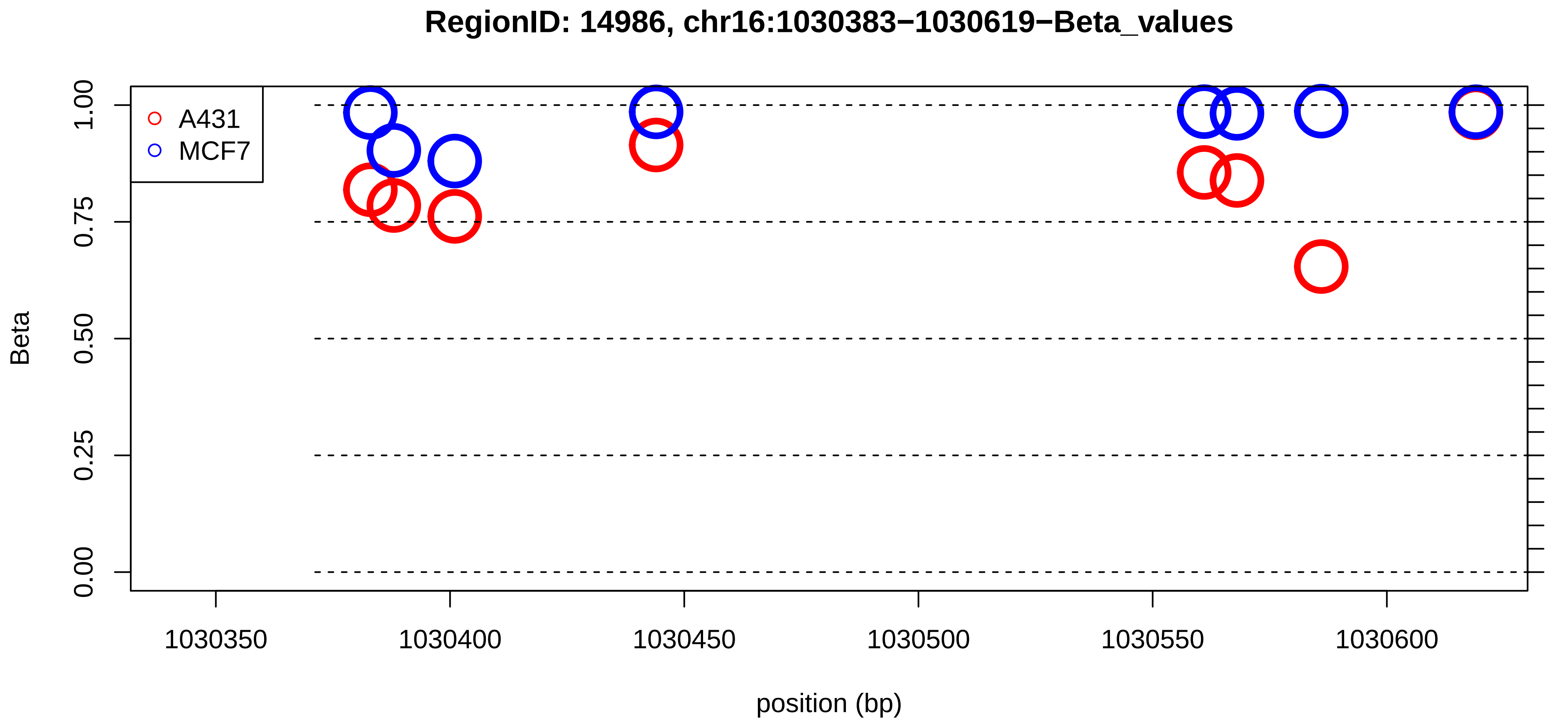

RegionID: 14986, chr16:1030383−1030619−Beta_values
A431
MCF7
Beta
0.00
0.25
0.50
0.75
1.00
1030350
1030400
1030450
1030500
1030550
1030600
position (bp)

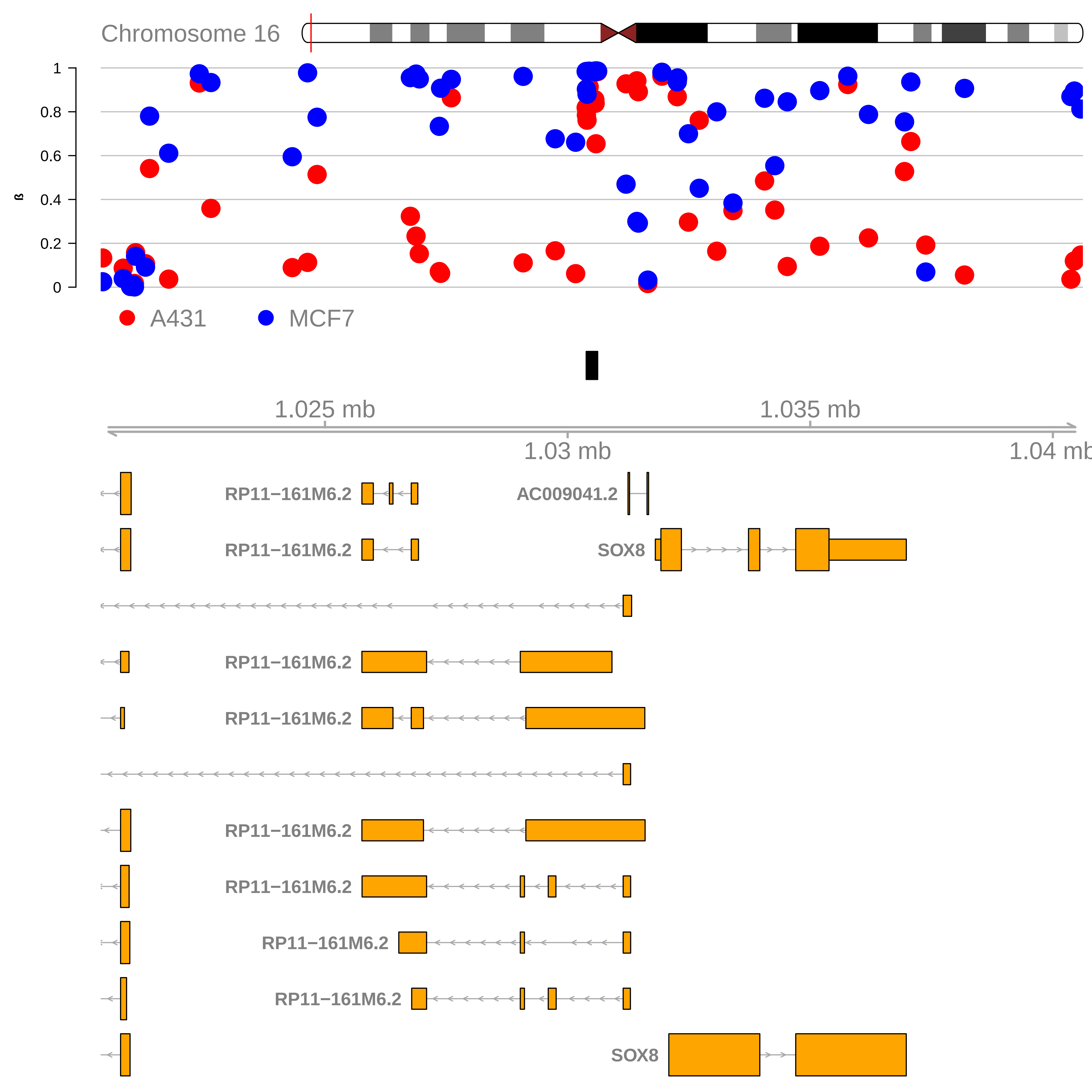

Chromosome 16
β
1
0.8
0.6
0.4
0.2
0
A431
MCF7
1.025 mb
1.035 mb
1.03 mb
1.04 mb
RP11−161M6.2
AC009041.2
SOX8
